# Siglec-8 Signals Through a Non-Canonical Pathway to Cause Human Eosinophil Death *In Vitro*

**DOI:** 10.3389/fimmu.2021.737988

**Published:** 2021-10-11

**Authors:** Daniela J. Carroll, Yun Cao, Bruce S. Bochner, Jeremy A. O’Sullivan

**Affiliations:** Division of Allergy and Immunology, Department of Medicine, Northwestern University Feinberg School of Medicine, Chicago, IL, United States

**Keywords:** Siglec, signaling/signaling pathways, CD11b, eosinophil, actin, Syk, Btk, cell death

## Abstract

Sialic acid-binding immunoglobulin-like lectin (Siglec)-8 is a glycan-binding receptor bearing immunoreceptor tyrosine-based inhibitory and switch motifs (ITIM and ITSM, respectively) that is selectively expressed on eosinophils, mast cells, and, to a lesser extent, basophils. Previous work has shown that engagement of Siglec-8 on IL-5–primed eosinophils causes cell death *via* CD11b/CD18 integrin–mediated adhesion and NADPH oxidase activity and identified signaling molecules linking adhesion, reactive oxygen species (ROS) production, and cell death. However, the proximal signaling cascade activated directly by Siglec-8 engagement has remained elusive. Most members of the Siglec family possess similar cytoplasmic signaling motifs and recruit the protein tyrosine phosphatases SHP-1/2, consistent with ITIM-mediated signaling, to dampen cellular activation. However, the dependence of Siglec-8 function in eosinophils on these phosphatases has not been studied. Using Siglec-8 antibody engagement and pharmacological inhibition in conjunction with assays to measure cell-surface upregulation and conformational activation of CD11b integrin, ROS production, and cell death, we sought to identify molecules involved in Siglec-8 signaling and determine the stage of the process in which each molecule plays a role. We demonstrate here that the enzymatic activities of Src family kinases (SFKs), Syk, SHIP1, PAK1, MEK1, ERK1/2, PLC, PKC, acid sphingomyelinase/ceramidase, and Btk are all necessary for Siglec-8–induced eosinophil cell death, with no apparent role for SHP-1/2, SHIP2, or c-Raf. While most of these signaling molecules are necessary for Siglec-8–induced upregulation of CD11b integrin at the eosinophil cell surface, Btk is phosphorylated and activated later in the signaling cascade and is instead necessary for CD11b activation. In contrast, SFKs and ERK1/2 are phosphorylated far earlier in the process, consistent with their role in augmenting cell-surface levels of CD11b. In addition, pretreatment of eosinophils with latrunculin B or jasplakinolide revealed that actin filament disassembly is necessary and sufficient for surface CD11b integrin upregulation and that actin polymerization is necessary for downstream ROS production. These results show that Siglec-8 signals through an unanticipated set of signaling molecules in IL-5–primed eosinophils to induce cell death and challenges the expectation that ITIM-bearing Siglecs signal through inhibitory pathways involving protein tyrosine phosphatases to achieve their downstream functions.

## Introduction

Siglecs (sialic acid-binding immunoglobulin-like lectins) are cell surface proteins found predominantly, but not exclusively, on the surface of immune cells and are characterized by their propensity to specifically bind sialic acid-containing structures. The human Siglec receptors are clustered into two subfamilies: a group of highly conserved Siglecs and the rapidly evolving CD33-related Siglecs, the latter including Siglec-8 (1). Siglec-8 is selectively expressed on the surface of human eosinophils and contains two cytoplasmic signaling motifs—an intracellular immunoreceptor tyrosine-based inhibitory motif (ITIM) and an immunoreceptor tyrosine-based switch motif (ITSM)—thought to be responsible for inhibitory signal transduction (2–4).

Previous work using IL-5–primed human eosinophils has shown that multimeric engagement of Siglec-8 induces caspase-independent, CD11b/CD18 integrin– and NADPH oxidase–dependent cell death associated with diminished mitochondrial membrane potential *via* a mechanism that involves the PI3K/Akt pathway and Rac1 activity (5–8). A rapidly mobilizable pool of intracellular CD11b/CD18, like the one trafficked to the cell membrane in response to Siglec-8 ligation (8), is known to exist in secretory vesicles in eosinophils but may also be associated with granule membranes as well (9). The source of the intracellular pool of CD11b/CD18 mobilized by Siglec-8 signaling has not been determined.

Several studies have demonstrated that the association of Siglec family members with ITAM-bearing receptors or the engagement of the Siglec family member alone leads to Src family kinase (SFK)-dependent phosphorylation of the ITIM and/or ITSM of the Siglec receptor, recruitment of Src-homology region 2 domain-containing phosphatases such as SHP-1 and SHP-2, and the subsequent inhibition of cell activation, consistent with canonical ITIM-based inhibitory signaling (10–14). Phosphorylation of the cytoplasmic signaling motifs and recruitment of SHP-1/2 have been further shown for other Siglecs through the use of the protein tyrosine phosphatase inhibitor pervanadate (15, 16), suggesting that ITIM-bearing Siglecs generally achieve their inhibitory effects through phosphatase recruitment. Although studies of Siglec-8 signaling in eosinophils with simultaneous IL-5 stimulation by Kano et al. have implicated SFKs such as Fgr in the cell death response (17), the involvement of protein tyrosine phosphatases typically associated with ITIM-bearing CD33-related Siglec signaling have not been explored in detail for Siglec-8.

The elucidation of the signaling cascade leading from Siglec-8 engagement to cell death of human eosinophils has been impeded by the relative lack of molecules with demonstrated roles in the process; a shortage of information about whether these molecules are activated by cytokine signaling, Siglec-8 ligation, integrin-mediated adhesion, or ROS-mediated events; and the inability to use genetic techniques to silence or knock out candidate molecules in mature human eosinophils. Nevertheless, delineating the process whereby Siglec-8 achieves the elimination of cytokine-primed eosinophils may lead to additional therapeutic options that could potentially circumvent the need for priming or reveal conditions under which Siglec-8 signaling, whether physiological or induced by therapeutic intervention, is unable to reduce eosinophil numbers.

To address this, we combined pharmacological inhibition with assays to measure Siglec-8 engagement–induced CD11b integrin upregulation at the cell surface, CD11b conformational activation, ROS production, and cell death to identify molecules necessary for the overall process and more precisely identify the sequence and types of events for which they are essential. We demonstrate that Siglec-8 induces a signaling cascade that is uncharacteristic for an ITIM-bearing receptor, involving the activities of SFKs, Syk, PI3K, SHIP1, Rac1, PLC, PKC, acid sphingomyelinase/ceramidase, PAK1, MEK1, ERK1/2, and Btk as well as actin rearrangement. No roles for c-Raf or SHP-1/2 were found in this pathway, although Siglec-8 appears to associate with SHP-2. Most of the identified molecules are involved in CD11b surface upregulation, whereas Btk is necessary later in the process for CD11b conformational activation. Actin filament disassembly is necessary and sufficient for CD11b upregulation and conformational activation but blocks ROS production. Thus, unlike the ITIM-dependent inhibitory effect of Siglec-8 antibody ligation on human mast cells (18), Siglec-8 engagement on IL-5–primed human eosinophils initiates a signaling pathway that is more typical of an immunoreceptor tyrosine-based activation motif (ITAM)-bearing receptor, in stark contrast with the expectation that an ITIM-bearing receptor and CD33-related Siglec, in particular, would exert its effect through SHP-1/2–mediated pathways.

## Materials and Methods

### Human Eosinophil Isolation and Culture With Various Pharmacologic Antagonists

Written informed consent for blood donation (up to 180 mL) was obtained using an institutional review board–approved protocol at the Northwestern University Feinberg School of Medicine. Eosinophils from mildly allergic and non-allergic donors were purified from peripheral blood using density gradient centrifugation, erythrocyte hypotonic lysis, and CD16 immunomagnetic negative selection (Miltenyi Biotec, San Diego, CA) as described (19). Purity and viability were consistently greater than 95% as determined by Siglec-8 staining and DAPI (ThermoFisher Scientific, Waltham, MA) exclusion (20). Cells were cultured in Roswell Park Memorial Institute (RPMI) 1640 medium with 10% FCS and antibiotics (all from ThermoFisher Scientific) as well as with or without 30 ng/ml rhIL-5 (R&D Systems, Minneapolis, MN) for 18–24 hr as indicated. To assess the involvement of various intracellular pathways in Siglec-8-mediated integrin upregulation, integrin activation, ROS production, or cell death, eosinophils were exposed to the following inhibitors/chemical agents at these concentrations unless otherwise indicated. OXSI-2 (Syk inhibitor; 667 nM) and SCH772984 (ERK1/2; 300 nM) were purchased from Cayman Chemical (Ann Arbor, MI). NSC-87877 (SHP-1/2), 3-AC (SHIP1; 10 µM), AS1938909 (SHIP1/2), GW5074 (c-Raf; 30 nM), nocodazole (microtubule polymerization inhibitor; 1 µM), latrunculin B (actin depolymerizing agent; 5 µM), jasplakinolide (F-actin stabilizing agent; 1 µM), and Luperox TBH70X *tert*-butyl hydroperoxide solution (oxidative stress inducer; 200 µM), and desipramine (disruptor of acid sphingomyelinase and acid ceramidase; 10 µM) were purchased from Millipore Sigma (Burlington, MA). R406 (Syk inhibitor), NSC-23766 (Rac1; 50 µM), PD98059 (MEK1/2; 30 µM), U0126 (MEK1/2; 10 µM), LY294002 (PI3K; 5 µM), PP1 (SFKs), SU6656 (SFKs), GF109203x (PKC; 5 µM), ibrutinib (Btk), and acalabrutinib (Btk; 10 nM) were purchased from Selleckchem (Houston, TX). AZ13705339 (PAK1/2 inhibitor; 300 nM), U73122 (phospholipase C inhibitor; 100 nM), and U73343 (negative control for U73122; 100 nM) were purchased from Tocris Bioscience (Bristol, UK). Cells were pre-incubated with each agent for 30 min at 37°C prior to addition of various stimuli, and the agent remained in the medium for the duration of the culture period.

### Determination of CD11b Upregulation, CD11b Activation, and Cell Death Following Siglec-8 Engagement

Eosinophils (2×10^5^ cells in 200 µL medium per condition) were cultured for 18–24 h at 37°C with 30 ng/ml rhIL-5, then anti-Siglec-8 monoclonal antibody (mAb; clone 2C4) or mouse IgG1 isotype control mAb was added to a final concentration of 2.5 µg/ml. In some experiments, F(ab′)_2_ fragments of anti-Siglec-8 mAb clone c2E2, which recognizes the same epitope as clone 2C4, were used at the same final concentration. F(ab′)_2_ fragments were generously provided by John Leung and Nenad Tomasevic (Allakos Inc., Redwood City, CA). CD11b upregulation and activation were assessed as previously described (8). Briefly, eosinophils were cultured for 2 hr after stimulation prior to washing, staining for CD11b surface expression (clone ICRF44; BD Biosciences, San Jose, CA) or CD11b conformational activation (clone CBRM1/5; BioLegend, San Diego, CA) in conjunction with the viability stain DAPI, and data were then collected on a BD LSR II flow cytometer. The analysis of changes in CD11b or activated CD11b levels on the cell surface of viable cells was performed using FlowJo software v10 (TreeStar, Ashland, OR). To assess cell death induction as a result of Siglec-8 engagement, the cells were treated with antibody for 18–24 h as previously described (8, 21), at which point cell death was assessed by flow cytometry after FITC-Annexin V (BioLegend) and DAPI labeling.

### ROS Detection

Eosinophil ROS production was measured using dihydrorhodamine 123 (DHR 123; ThermoFisher Scientific). Eosinophils were cultured with rhIL-5 in an identical manner to the cell death experiments mentioned above and were then transferred to 5-ml polystyrene round-bottom tubes (2×10^5^ eosinophils in 200 µL per sample). After pretreatment with the indicated pharmacological inhibitors, the cells were loaded with DHR 123 for 15 min at 37°C and then the indicated mAb was added at a final concentration of 2.5 µg/ml as before. After 120 min, the cells were washed, stained with DAPI, and analyzed by flow cytometry. Levels of ROS generation were normalized to maximum ROS production induced by the anti-Siglec-8 mAb within each experiment as done previously (8).

### Detection of Phosphorylated Signaling Proteins

Phosphorylated signaling proteins were measured by either traditional western blot or automated protein separation and immunodetection. For traditional western blotting, eosinophils (5×10^5^ per condition) were incubated at 37°C with anti-Siglec-8 or isotype control mAb for durations of time. Next, 2x Laemmli buffer (Bio-Rad, Hercules, CA) was used to lyse the eosinophils and isolate protein, which was electrophoresed through 10% mini-PROTEAN TGX precast gels and transferred to Immun-Blot low-fluorescence PVDF membranes using the Trans-Blot SD semi-dry transfer cell (Bio-Rad), according to the manufacturer’s guidelines. After blocking with Blocking Buffer for Fluorescent Western Blotting (MB-070, Rockland, Limerick, PA), membranes were incubated overnight with various primary antibodies (used at 1:500–1:1000 dilutions in blocking buffer) against phosphotyrosine (clone 4G10; Millipore Sigma), total Btk (Cell Signaling Technology, Danvers, MA), phospho-Btk (pTyr551; BD Biosciences), and total beta-actin (Thermo Scientific). Antibody binding was detected with IRDye 680RD- or 800CW-conjugated secondary antibodies (LI-COR, Lincoln, NE) using the Odyssey Imaging System (LI-COR). Automated protein separation and chemiluminescent immunodetection was performed using the Jess instrument (ProteinSimple, San Jose, CA). Eosinophils (2×10^5^ per sample) were lysed in RIPA buffer supplemented with protease and phosphatase inhibitors. Antibodies against phospho-ERK1/2 (pThr202, pTyr204), phospho-SFK (pTyr419), total Src (Cell Signaling Technology), and total ERK1/2 (ProteinSimple) were used in these assays. Data were analyzed using Compass for SW (v4.1.0; ProteinSimple).

### Statistical Analysis

Data are presented as mean ± standard deviation unless otherwise indicated. Statistical significance was determined by two-way ANOVA and Dunnett (for comparisons with a single [vehicle] control) or Šidák (for pairwise comparisons at a given pharmacological inhibitor concentration) corrections for multiple comparisons using GraphPad Prism 6.0e. Statistical differences were considered significant at p < 0.05.

## Results

### Functional Role for SHIP1 but Not SHP-1/2 in Siglec-8–Mediated Cell Death

**Figure 1:**
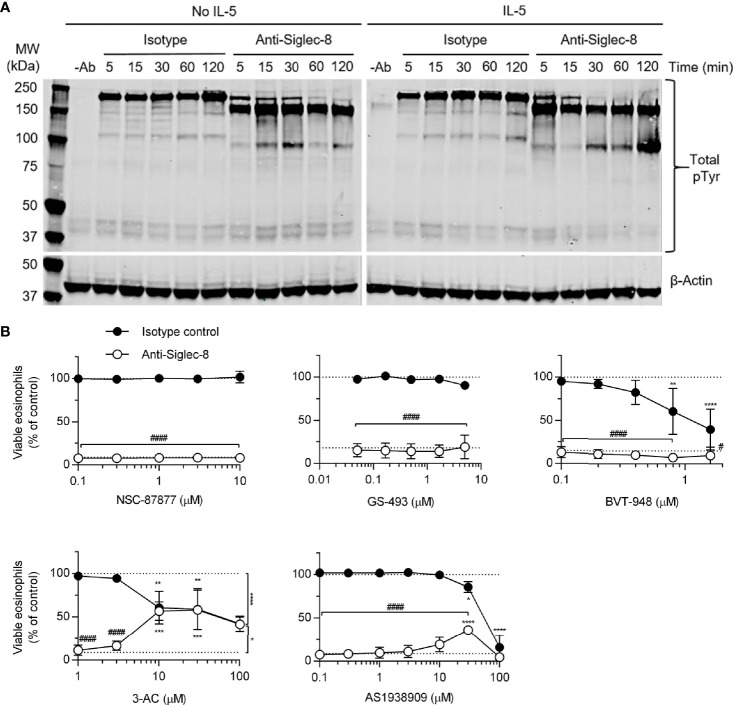
SHIP1 activity is necessary for Siglec-8–induced eosinophil cell death. **(A)** Eosinophils were treated with anti-Siglec-8 (clone 2C4) or isotype control mAb (IgG1) for the indicated durations in the absence of IL-5 (No IL-5) or after being primed in the presence of 30 ng/ml IL-5 for 18–24 hr. The cells were then lysed, and the lysates separated by SDS-PAGE and transferred to a PVDF membrane. Tyrosine-phosphorylated proteins were detected using a phosphotyrosine-specific antibody. Blots are representative of three independent experiments. **(B)** IL-5–primed eosinophils were pretreated with pharmacological inhibitors of SHP-1/2 (NSC-87877, GS-493, BVT-948), SHIP1 (3-AC), or SHIP2 and SHIP1 (AS1938909) at the indicated concentrations for 30 min prior to treatment with anti-Siglec-8 (open circles) or isotype control mAb (filled circles) for 18–24 hr. Cell viability was then assessed by annexin V and DAPI staining by flow cytometry and normalized to the cell viability measured in untreated samples. Dotted lines indicate viability levels with no pharmacological inhibition. Data represent means ± standard deviations of 3 independent experiments. *p < 0.05, **p < 0.01, ***p < 0.001, ****p < 0.0001 relative to within–antibody stimulation group vehicle control sample. ^#^p < 0.05, ^####^p < 0.0001 relative to isotype control sample at the same pharmacological inhibitor concentration.

The cellular consequences of antibody engagement of Siglec-8 on eosinophils depends on whether the eosinophil has been primed by stimulation with cytokines such as IL-5, GM-CSF, or IL-33 (6–8, 21): primed eosinophils—but not unprimed eosinophils—undergo cell death following Siglec-8 engagement. In order to broadly characterize signaling events downstream of Siglec-8 ligation in cytokine-primed or unprimed eosinophils, tyrosine-phosphorylated proteins were visualized by western blot after several durations of antibody treatment (**Figure 1A**). There was an acceleration of tyrosine phosphorylation events due to Siglec-8 engagement in IL-5–primed eosinophils and a band below 100 kDa in molecular weight that becomes particularly prominent at 120 min in these cells. Notably, there is no apparent protein dephosphorylation relative to baseline—the untreated control lane—as one might expect from the inhibition of ITAM-mediated signaling by an ITIM-bearing receptor. To more directly assess the roles played by protein and lipid phosphatases associated with ITIM-mediated signaling in Siglec-8 function, eosinophils were pretreated with the SHP-1/2 inhibitors NSC-87877, GS-493, and BVT-948; the SHIP1 inhibitor 3-AC; or the SHIP2 inhibitor AS1938909, which also acts on SHIP1 at higher concentrations. While the inhibitors of SHP-1/2 and SHIP2 did not affect the extent of cell death at concentrations near the IC_50_ values for these molecules, 3-AC and AS1938909 prevented Siglec-8–induced cell death at concentrations that would be expected to inhibit SHIP1 function (3-AC: IC_50,SHIP1_ ≈ 10 µM (22); AS1938909: IC_50,SHIP2_ = 0.57 µM, IC_50,SHIP1_ = 21 µM (23); **Figure 1B**). However, Siglec-8 was not detected in SHIP1 immunoprecipitates of lysates of eosinophils that had been treated with sodium orthovanadate (**Supplementary Figure 1**), suggesting that SHIP1 is activated indirectly or that this association is transient. Rather, there was an association observed between Siglec-8 and SHP-2, the significance of which is unclear.

### SFK, Syk, and Btk Activities Are Necessary for Siglec-8 Function

**Figure 2:**
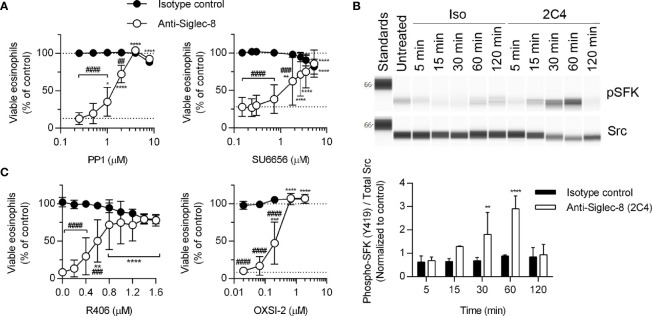
SFKs and Syk are necessary components of the Siglec-8 signaling cascade. **(A)** Eosinophils were primed with IL-5 and pretreated with pharmacological inhibitors that act on SFKs (PP1, SU6656) at the indicated concentrations for 30 min prior to treatment with anti-Siglec-8 (open circles) or isotype control mAb (filled circles) for 18–24 hr. Cell viability was then assessed by annexin V and DAPI staining by flow cytometry and normalized to that of untreated samples. Dotted lines indicate viability levels with no pharmacological inhibition. Data represent means ± standard deviations of 3 and 5 independent experiments, respectively. **(B)** Primed eosinophils were incubated with anti-Siglec-8 (2C4) or isotype control mAb (IgG1) for the indicated durations (in minutes) prior to lysis and the subsequent separation and detection of phospho-SFK (pY419) and total Src. Upper panel: representative western blot. Lower panel: quantified band intensities representing the means ± standard deviations of 4 independent experiments. Band intensities were normalized to those of untreated control samples. **p < 0.01, ****p < 0.0001 vs. isotype control samples at the same time point. **(C)** Primed eosinophils were pretreated with the Syk inhibitors R406 or OXSI-2 at the indicated concentrations for 30 min prior to treatment with anti-Siglec-8 (open circles) or isotype control mAb (filled circles) for 18–24 hr. Cell viability was assessed as in **(A)**. Data represent means ± standard deviations of 3 independent experiments. **(A, C)** *p < 0.05, **p < 0.01, ***p < 0.001, ****p < 0.0001 relative to within–antibody stimulation group vehicle control sample. ^##^p < 0.01, ^###^p < 0.001, ^####^p < 0.0001 relative to isotype control sample at the same pharmacological inhibitor concentration.

The proximal signaling molecules involved in Siglec-8 signaling have been elusive. Although Src family kinases (SFKs) have been implicated in Siglec-8–induced eosinophil death (17), SFK activation loop phosphorylation has not been demonstrated and dose-response curves for SFK inhibitors are lacking. In order to confirm a role for SFKs in this process, eosinophils were pretreated with two additional SFK inhibitors, PP1 and SU6656, at various concentrations and SFK phosphorylation at Y419 was evaluated by western blot following Siglec-8 antibody engagement. Indeed, both inhibitors prevented Siglec-8–induced cell death at concentrations consistent with targeting SFKs selectively (24, 25) (**Figure 2A**). Additionally, activating phosphorylation (pY419) of an SFK of approximately 58 kDa was observed following Siglec-8 ligation at statistically significant levels at 30 and 60 minutes (**Figure 2B**).

In addition to the overall pattern of protein tyrosine phosphorylation in response to Siglec-8 ligation (**Figure 1A**), previous publications indicate the Siglec-8 signaling pathway does not resemble a typical ITIM-mediated pathway and instead incorporates signaling molecules typically associated with ITAM-mediated signaling in a process that involves the upregulation and activation of CD11b and the generation of ROS (8, 26), although it is unclear which molecules act upstream of these cellular events and which act only downstream. Syk family proteins are involved in ITAM-mediated signaling downstream of SFKs, and Syk has been linked to integrin signaling and function previously (27–30). Therefore, we employed two Syk inhibitors, R406 and OXSI-2, to determine whether its enzymatic activity is necessary for Siglec-8-induced cell death. At concentrations appropriate for their selective inhibitory effects on Syk, both inhibitors blocked cell death (**Figure 2C**), indicating an essential role for this molecule in the pathway.

**Figure 3:**
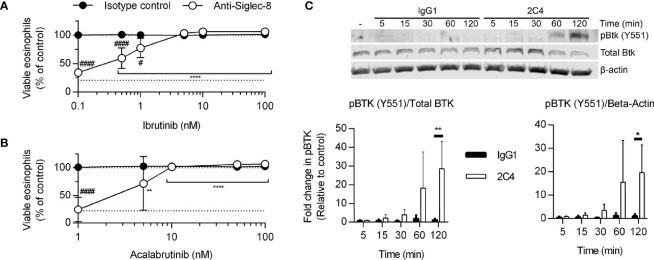
Btk is activated late in the Siglec-8 signaling pathway and is necessary for Siglec-8–induced eosinophil cell death. Human eosinophils were stimulated with IL-5 and pretreated with the indicated concentrations of the Btk inhibitors ibrutinib **(A)** or acalabrutinib **(B)** for 30 min prior to treatment with anti-Siglec-8 (open circles) or isotype control (filled circles) for 18–24 h. Eosinophil viability was then assessed by annexin V/DAPI staining as before. Dotted lines indicate viability levels with no pharmacological inhibition. Plots represent mean normalized eosinophil viability ± standard deviations for 3 independent experiments. **p < 0.01, **** p < 0.0001 relative to within–antibody stimulation group vehicle control sample. ^#^p < 0.05, ^####^p < 0.0001 relative to isotype control sample at the same pharmacological inhibitor concentration. **(C)** Eosinophils were treated with anti-Siglec-8 mAb (2C4, white columns), isotype control mAb (IgG1, black columns), or left untreated for the indicated durations before lysis and separation of proteins by SDS-PAGE. Immunoblotting for phospho-Btk, total Btk, and β-actin was performed. The blot is representative of 3 independent experiments. Quantitation of phospho-Btk relative to β-actin or total Btk normalized to the untreated eosinophils is shown. The data represent means ± standard deviations for 3 independent experiments. *p < 0.05; **p < 0.01.

Because the Tec family kinase Bruton’s tyrosine kinase (Btk) has been found to play a role in integrin activation (31), we tested the effects of the Btk inhibitors ibrutinib and acalabrutinib on Siglec-8-induced eosinophil death. At concentrations consistent with their reported IC_50_ values for Btk, each inhibitor prevented Siglec-8 engagement-induced cell death (**Figures 3A, B**). Furthermore, phosphorylation and activation of Btk was observed in a Siglec-8 engagement–specific manner, which reached statistical significance following 120 min of antibody treatment (**Figure 3C**), likely indicating a role for Btk later in the cell death process.

### MEK1, ERK1/2, and PAK1 Are Necessary for Siglec-8 Function

**Figure 4:**
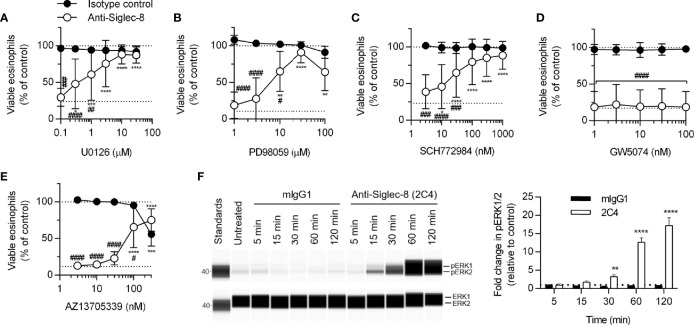
The activities of MEK1, ERK1/2, and PAK1 are necessary for Siglec-8–induced eosinophil cell death. Peripheral blood eosinophils were stimulated with 30 ng/ml IL-5 overnight and pretreated with the indicated concentration of the MEK1/2 inhibitors U0126 **(A)** or PD98059 **(B)**, ERK1/2 inhibitor SCH772984 **(C)**, c-Raf inhibitor GW5074 **(D)**, or PAK1/2 inhibitor AZ13705339 **(E)** for 30 min prior to 18–24-h treatment with anti-Siglec-8 (open circles) or isotype control mAb (filled circles). Cell viability was then assessed by annexin V/DAPI staining by flow cytometry. Dotted lines indicate viability levels with no pharmacological inhibition for inhibitors plotted on a logarithmic scale. Plots represent the mean percentage of viable eosinophils normalized to the viability of untreated eosinophils ± standard deviations for 3 independent experiments. *p < 0.05, **p < 0.01, ***p < 0.001, ****p < 0.0001 relative to within–antibody stimulation group vehicle control sample. ^#^p < 0.05, ^##^p < 0.01, ^###^p < 0.001, ^####^p < 0.0001 relative to isotype control sample at the same pharmacological inhibitor concentration. **(F)** IL-5–primed eosinophils were treated with anti-Siglec-8 mAb or isotype control mAb (mIgG1) for the indicated durations prior to lysis and detection of phospho- (pT202/pY204) or total ERK1/2 using an automated protein separation and immunodetection platform. Blot is representative and quantified data represent the means and standard deviations of three independent experiments. **p < 0.01; ****p < 0.0001 vs. isotype control at the same time point.

MEK1/2 have previously been shown to be necessary for Siglec-8 engagement-induced eosinophil death with short-term IL-5 treatment (26); however, whether MEK1 or MEK2 is the critical molecule or whether the upstream and downstream signaling molecules c-Raf and ERK1/2 are also necessary have not been determined. We first demonstrated that, with longer-term IL-5 treatment, the dual MEK1/2 inhibitor U0126 also prevents Siglec-8 engagement–induced cell death (**Figure 4A**) before using another inhibitor, PD98059, that can be used to discern between MEK1 and MEK2 activity on the basis of its different IC_50_ values for these proteins. The use of this inhibitor indicated it is likely MEK1 activity—and not that of MEK2, which is inhibited at lower drug concentrations—that is necessary for cell death induction in this pathway (**Figure 4B**). In addition, the use of the ERK1/2 and c-Raf activity–selective inhibitors (SCH772984 and GW5074, respectively) demonstrate that downstream ERK1/2 activity is indeed necessary for cell death, although no role for c-Raf was observed (**Figures 4C, D**). In lieu of c-Raf, p21 (Rac1)–activated kinase 1 (PAK1) is capable of activating MEK1 in pathways that result in degranulation of human NK cells and cell death in human neutrophils but does not appreciably activate MEK2 (32–34). In addition, we have previously published that the activation of the molecule immediately upstream of PAK1, Rac1, is crucial for Siglec-8 engagement-induced eosinophil death (8). Use of the PAK1/2-selective inhibitor AZ13705339 prevented Siglec-8 engagement–induced eosinophil death at a concentration similar to the published IC_50_ of the compound for PAK1 [**Figure 4E**; 59 nM (35)]. Activating phosphorylation of ERK1/2 (pT202/pY204) was observed only in eosinophils stimulated through Siglec-8 starting after 30 minutes of stimulation (**Figure 4F**), confirming that ERK1/2 is involved downstream of Siglec-8 ligation and not IL-5 receptor signaling under these conditions.

### The PLC-PKC Signaling Axis and the Sphingolipid Metabolic Pathway Are Required for Siglec-8 Function

**Figure 5:**
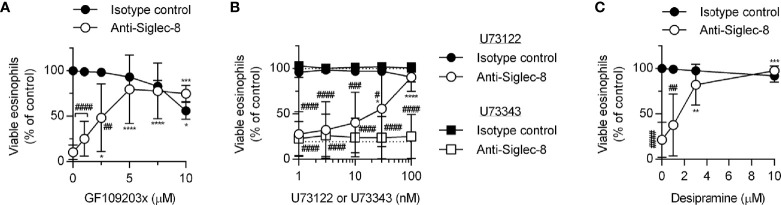
The PLC-PKC signaling axis and sphingomyelin metabolism are necessary for Siglec-8–induced eosinophil cell death. Peripheral blood eosinophils were stimulated with 30 ng/ml IL-5 overnight and pretreated with the indicated concentration of the PKC inhibitor GF109203x **(A)**, the PLC inhibitor U73122 (circle symbols) or pharmacologically inactive analog U73343 (square symbols) **(B)**, or the functional acid sphingomyelinase and acid ceramidase inhibitor desipramine **(C)** for 30 min prior to 18–24-h treatment with anti-Siglec-8 (open symbols) or isotype control mAb (filled symbols). Cell viability was then assessed by annexin V/DAPI staining by flow cytometry. Dotted lines indicate viability levels with no pharmacological inhibition for inhibitors plotted on a logarithmic scale. Plots represent the mean percentage of viable eosinophils normalized to the viability of untreated eosinophils ± standard deviations for 3 independent experiments. *p < 0.05, **p < 0.01 ***p < 0.001, ****p < 0.0001 relative to within–antibody stimulation group vehicle control sample. ^#^p < 0.05, ^##^p < 0.01, ^###^p < 0.001, ^####^p < 0.0001 relative to isotype control sample at the same pharmacological inhibitor concentration.

Because PKC activity has been implicated in the regulation of integrin adhesion and signaling, we hypothesized that PKC may play a similar role downstream of Siglec-8 engagement. Treatment of eosinophils with the PKC-selective inhibitor GF109203x blocked Siglec-8–induced cell death (**Figure 5A**), although the observed IC_50_ of this effect may indicate a role for novel or atypical PKC isozymes rather than the conventional PKC isozymes for which this compound is a more potent inhibitor. To help clarify which PKC class is likely involved in Siglec-8 signaling, inhibition of phospholipase C (PLC), a family of enzymes responsible for producing the conventional and novel PKC isozyme–activating second messenger diacylglycerol, was assessed within the cell death assay. Use of the PLC inhibitor U73122 revealed that PLC activity is essential for Siglec-8 engagement–induced eosinophil death, and, as anticipated, the structurally related but inactive analog U73343 did not impact cell viability (**Figure 5B**). One potential link between PKC signaling and the PAK1-MEK1-ERK1/2 signaling pathway is that PKCδ phosphorylates and activates acid sphingomyelinase (36, 37), which acts in the first step of the process in the conversion of sphingomyelin to sphingosine, a lipid that promotes the activation of PAK1 (38, 39). Alternatively, or additionally, PKC may promote integrin conformational activation through the activation of the small GTPase Rap1 (40). To test the first possibility, desipramine was used to destabilize and cause the degradation of acid sphingomyelinase and acid ceramidase (41, 42), the lysosomal enzymes necessary to convert sphingomyelin to sphingosine, to determine whether this metabolic pathway is necessary for Siglec-8 function. Desipramine treatment prevented Siglec-8 ligation-induced eosinophil death at a concentration consistent with this effect (**Figure 5C**).

### Actin Cytoskeletal Dynamics Are Necessary for Siglec-8-Induced Cell Death

**Figure 6:**
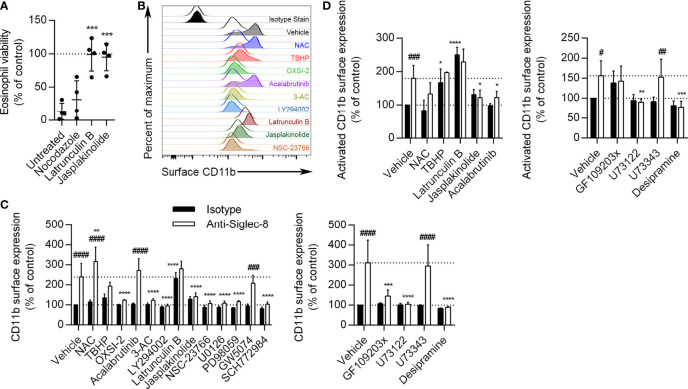
Actin depolymerization is necessary and sufficient for CD11b surface upregulation and activation, and Btk is involved in CD11b activation but not upregulation. **(A)** Eosinophils were pretreated with the indicated disruptors of cytoskeletal dynamics for 30 min prior to treatment with anti-Siglec-8 or isotype control mAb. After 18–24 h, eosinophil cell viability was assessed by annexin V/DAPI staining as before, and normalized to the isotype control sample within each treatment. Individual results, means, and standard deviations are shown for 4 independent experiments. ***p < 0.0001 relative to vehicle control–pretreated eosinophils. **(B)** Following pretreatment with the indicated agents for 30 min and treatment with anti-Siglec-8 (filled histograms) or isotype control mAb (empty histograms) for 120 min, surface expression of CD11b was assessed by flow cytometry on live (DAPI-negative) eosinophils. Isotype control mAb-stained samples are included for reference. Histograms are representative of 3 independent experiments. **(C)** CD11b expression levels on eosinophils treated with anti-Siglec-8 (white columns) or isotype control mAb (black columns) were quantified and normalized to those of control eosinophils. Data represent means ± standard deviations of 3 independent experiments. **p < 0.01, ***p < 0.001, ****p < 0.0001 relative to within–antibody stimulation group vehicle control sample. ^###^p < 0.001, ^####^p < 0.0001 relative to within–pretreatment group isotype control sample. **(D)** Following pretreatment with the indicated agents for 30 min and treatment with anti-Siglec-8 (white columns) or isotype control mAb (black columns) for 120 min, conformational activation of CD11b on live (DAPI-negative) eosinophils was measured by flow cytometry. Activated CD11b levels were normalized to those found on control eosinophils. Data represent means ± standard deviations of 4 independent experiments. *p < 0.05, **p < 0.01, ***p < 0.001, ****p < 0.0001 relative to within–antibody stimulation group vehicle control sample. ^#^p < 0.05, ^##^p < 0.01, ^###^p < 0.001 relative to within–pretreatment group isotype control sample.

Prior work has shown that many of the proteins phosphorylated as a result of Siglec-8 ligation on IL-5–primed eosinophils—and not on unprimed eosinophils—are involved in cytoskeletal rearrangement, including Formin-1, L-plastin, Talin-2, Wiskott-Aldrich syndrome protein family member 3, PDZ and LIM domain protein 2, Swiprosin-1/EF-hand domain–containing protein D2, and microtubule-associated proteins 1A/1B light chain 3C (8). In order to assess whether cytoskeletal rearrangement is necessary for Siglec-8 function in cytokine-primed eosinophils, we disrupted microtubules with nocodazole, blocked actin filament formation with latrunculin B, and promoted actin filament polymerization with jasplakinolide prior to antibody treatment. We found that disruption of the actin cytoskeleton but not microtubules prevented cell death, suggesting that both polymerization and depolymerization of actin filaments are necessary for Siglec-8 function (**Figure 6A**). These compounds exerted similar effects on Siglec-8 endocytosis in a previous study (43), leaving open the possibility that Siglec-8 endocytosis is necessary to enable downstream signaling leading to cell death.

### Most of the Identified Critical Signaling Molecules Are Necessary for Siglec-8 Ligation–Induced CD11b Upregulation

While roles for actin cytoskeletal dynamics and a number of signaling molecules have been established in the cell death pathway induced by Siglec-8 ligation, it is not clear at what point in the process each molecule acts. For example, these molecules may be involved in Siglec-8 signaling itself or may only be activated following integrin clustering or ROS production. To begin to establish an order of signaling events, we pretreated eosinophils with pharmacological inhibitors that have been effective in blocking cell death and analyzed their effects on earlier cellular events, namely the surface upregulation and conformational activation of CD11b integrin and ROS production. The inhibitors were used at the lowest concentrations that provided at least 80% protection from Siglec-8 engagement–induced cell death in the previous assays, as listed in the Methods. The concentration of the negative control U73343 was matched to that of U73122, and GW5047 was used at 30 nM. At 120 min after Siglec-8 ligation, CD11b surface expression on eosinophils increased about 2.5–3-fold relative to initial levels (**Figures 6B, C**). Consistent with the previously published finding that antibody blockade of CD11b/CD18 integrin subunits prevents ROS production downstream of Siglec-8 ligation, placing integrin involvement upstream of NADPH oxidase activation (8), the ROS scavenger N-acetylcysteine (NAC) did not prevent CD11b surface upregulation. In fact, NAC slightly increased the upregulation of CD11b at the cell surface; however, treatment with *tert*-butyl hydroperoxide (TBHP), a cell-permeable inducer of oxidative stress, did not statistically significantly alter CD11b upregulation. The induction of actin depolymerization with latrunculin B increased CD11b levels with or without Siglec-8 ligation to levels statistically indistinguishable from those induced by Siglec-8 ligation without pretreatment. Conversely, the promotion of actin filament formation *via* jasplakinolide treatment blocked the upregulation of CD11b at the cell surface. The pharmacological agents acting on Syk, SHIP1, PI3K, Rac1, MEK1, ERK1/2, PKC, PLC, and acid sphingomyelinase/ceramidase all prevented cell surface upregulation of CD11b (**Figures 6B, C**). These results demonstrate that integrin-mediated adhesion, which occurs downstream, does not underlie the involvement of these molecules more commonly associated with activating pathways. To assess the pathway in the absence of any inadvertent engagement of activating Fc receptors, we used anti-Siglec-8 F(ab′)_2_ fragments to engage the receptor. This engagement induced similar levels of cell death relative to intact mAb, in agreement with our previous results (8), *via* a pathway that was similarly dependent on the activities of Syk, PI3K, and PLC (**Supplementary Figure 2**), indicating that this pathway is genuinely the result of Siglec-8 engagement. In contrast, the Btk inhibitor acalabrutinib did not significantly affect the upregulation of CD11b, suggesting that Btk is involved at a later stage of this process. As expected, the inhibitor of c-Raf, GW5074, and the analog control for the PLC inhibitor, neither of which prevented Siglec-8 ligation-induced cell death, also did not prevent CD11b upregulation. Rapidly mobilizable intracellular stores of CD11b have been described on the membranes of secretory vesicles human eosinophils and may also occur on granule membranes (9).

### Btk Activity Is Necessary for Siglec-8 Ligation–Induced CD11b Activation

Focusing on the roles of ROS, actin dynamics, and Btk, we also examined the conformational activation of CD11b as the likely next step in the process. Following 120 min of Siglec-8 ligation, activated CD11b expression at the cell surface was found to approximately double (**Figure 6D**). The activation of CD11b was blocked by promotion of F-actin polymerization (jasplakinolide) as well as by the inhibition of Btk (acalabrutinib), suggesting that Btk is involved in integrin activation but not upregulation. Furthermore, the prevention of actin polymerization by latrunculin B and the induction of oxidative stress by TBHP caused CD11b activation even in the absence of Siglec-8 antibody engagement. PLC and acid sphingomyelinase/ceramidase inhibition also prevented CD11b activation (**Figure 6D**). Activated CD11b levels following GF109203x pretreatment did not statistically change in response to Siglec-8 engagement, although these levels also did not statistically differ from those observed without pretreatment.

### Actin Filament Formation Is Essential for Siglec-8 Ligation–Induced ROS Production

**Figure 7:**
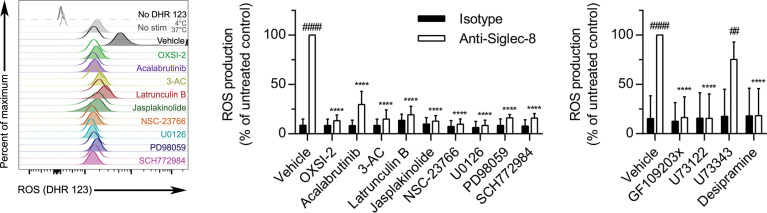
In addition to molecules involved in CD11b surface upregulation and activation, actin polymerization is necessary for Siglec-8–induced ROS production. Following pretreatment with the indicated agents for 30 min and treatment with anti-Siglec-8 (filled histograms) or isotype control mAb (empty histograms) for 120 min, ROS production was assessed by flow cytometry on live (DAPI-negative) eosinophils using DHR 123. Controls include samples lacking the DHR 123 probe, or those with the stain but maintained at 4°C or 37°C without stimulation (No stim). DHR 123 fluorescence was normalized to that of anti-Siglec-8 mAb–stimulated eosinophils that were not treated with any pharmacological inhibitor. Data represent means ± standard deviations of 3 independent experiments. ****p < 0.0001 relative to within–antibody stimulation group vehicle control sample. ^##^p < 0.01, ^####^p < 0.0001 relative to within–pretreatment group isotype control sample.

Siglec-8 engagement–induced eosinophil death is mediated by ROS *via* an as-yet undefined pathway (5), although there is evidence that the MEK/ERK pathway may additionally be involved after ROS generation (26). To determine whether these essential signaling molecules first act upstream or downstream of ROS production, the effect of each pharmacological agent on ROS production stimulated by anti-Siglec-8 mAb treatment was assessed. All of the inhibitors that prevented CD11b surface upregulation or activation also prevented ROS production (**Figure 7**), consistent with our previous study demonstrating the necessity of CD11b/CD18 integrin-mediated adhesion for ROS production in this pathway (8). In addition, latrunculin B, which promoted the expression and activation of CD11b at the cell surface even in the absence of Siglec-8 ligation, also blocked ROS production *via* the Siglec-8 signaling pathway, suggesting that F-actin polymerization plays a critical role between integrin activation and NADPH oxidase activation. Similarly, inhibitors of PKC, PLC, and acid sphingomyelinase/ceramidase also prevented Siglec-8 engagement–induced ROS production, whereas the negative control for PLC inhibition, U73343, as anticipated, had no apparent effect.

## Discussion

**Figure 8:**
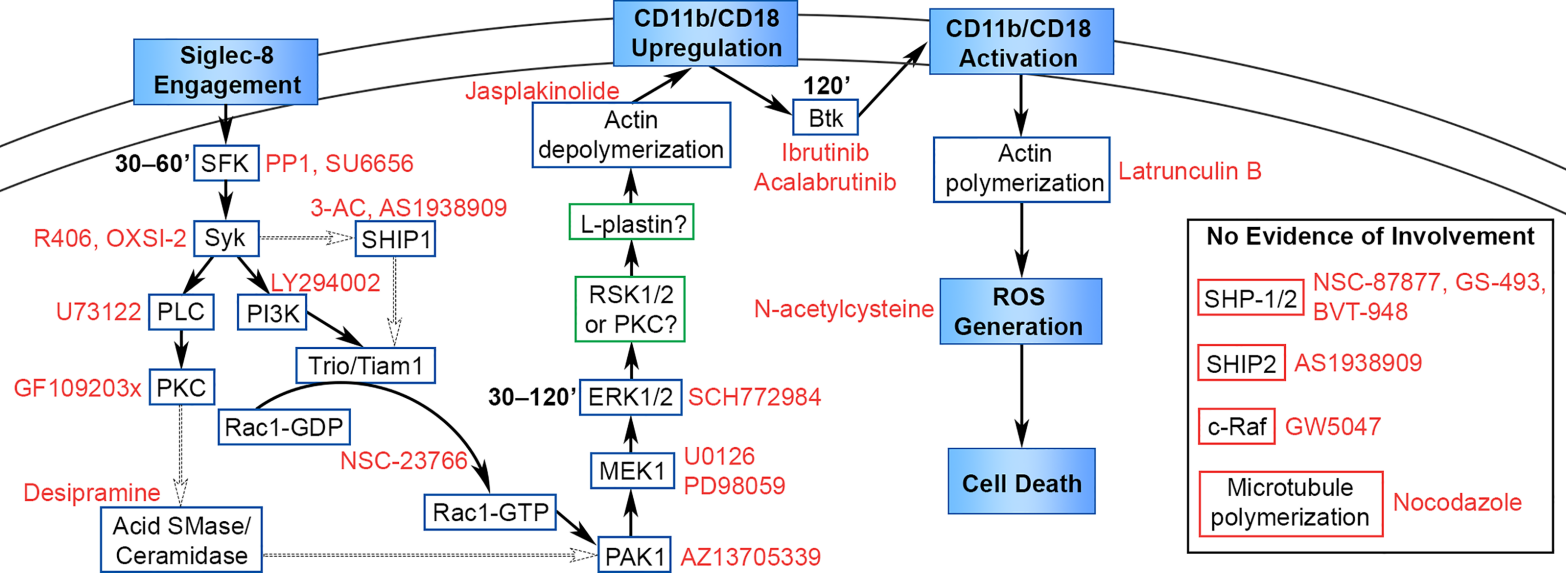
Proposed signaling pathway leading from Siglec-8 engagement to eosinophil cell death. Cellular events that occur in the cell death process are named in filled boxes. Positively identified signaling molecules are placed in boxes outlined in blue, candidate molecules that may be involved in the pathway are placed in boxes outlined in green, and molecules that do not appear to be involved in the pathway are placed in boxes outlined in red. Pharmacological inhibitors are named in red text adjacent to their molecular target, and the timing of signaling molecule activation is added in bold text adjacent to SFK, ERK1/2, and Btk. The data in this study identify and place molecules necessary in this pathway upstream or downstream of the named cellular events in the diagram. The precise sequence of molecules is hypothetical based on the literature, with well-supported interactions indicated with solid arrows and possible interactions indicated with dotted arrows.

Siglec-8 ligation on cytokine-primed human eosinophils induces cell death in an ROS-dependent manner and represents a potentially important pathway *in vivo* whereby eosinophil numbers may be regulated physiologically or modulated therapeutically (5, 6). More recently, we demonstrated that this process is dependent on cell adhesion through CD11b/CD18 integrin, which is essential for ROS production (8). However, the proximal signaling pathway initiated by Siglec-8 engagement that leads to integrin upregulation and activation had not previously been elucidated. We show here that antibody engagement of Siglec-8 on IL-5–primed eosinophils initiates a signaling cascade involving SFKs, Syk, PI3K, SHIP1, Rac1, PLC, PKC, acid sphingomyelinase/ceramidase, PAK1, MEK1, ERK1/2, and actin filament disassembly that acts to upregulate cell-surface levels of CD11b (**Figure 8**). There was no evidence of c-Raf, SHIP2, microtubule polymerization, or SHP-1/2 involvement in this pathway, despite an apparent association between SHP-2 and Siglec-8 following orthovanadate treatment. Furthermore, Btk activity and actin polymerization are also necessary for Siglec-8–induced cell death but are involved later in the process to induce CD11b conformational activation and promote ROS production, respectively.

Upregulation of CD11b on the surface of human eosinophils, which is associated with cell activation, is induced by stimulation with IL-3, GM-CSF, IL-5, IL-33, or eotaxin-1, among others, and is observed on eosinophils obtained from patients during the allergy season or in response to segmental or whole-lung antigen challenge [reviewed in (44)]. However, the pathway leading to this elevation in surface CD11b has not been fully delineated in eosinophils. More is known about the upregulation of CD11b on neutrophils, in which CD11b is transported to the cell membrane generally *via* the exocytosis of secretory vesicles upon stimulation, although secondary (specific) and tertiary (gelatinase) granules also augment surface CD11b levels upon their exocytosis (45, 46). Intracellular stores of CD11b in human eosinophils also appear to be primarily found on the membranes of secretory vesicles (9) and may therefore be transported to the cell surface *via* a similar pathway. The lack of eosinophil degranulation upon Siglec-8 engagement further indicates that this pool of CD11b molecules likely originated from secretory vesicles (8).

In parallel with the findings of this study, Syk has been found to be essential for the surface upregulation of CD11b on mouse neutrophils in response to stimulation with opsonized *S. aureus* in a genetic knockout model (29). Due to the lack of signaling motifs associated with Syk recruitment and activation in the cytoplasmic tail of Siglec-8 (4), it is possible that Siglec-8 interacts with signaling adapter molecules, such as DAP12, as has been reported for Siglecs-14, -15, and -16 in humans (47–49) or Siglec-H in mice (50), or with other cell-surface receptors to initiate this downstream signaling cascade. ITAM-mediated signaling proceeds through SFKs to activate Syk signaling. Our studies augment the evidence of the necessity of SFK activity in this pathway and reduce the likelihood of a common unintended target by using two additional selective inhibitors across a range of concentrations and demonstrating activating phosphorylation of an SFK downstream of Siglec-8 ligation.

Syk, shown here to be necessary for the initial upregulation of CD11b at the cell surface of eosinophils, has been shown to be necessary for the activation of PI3K signaling upon B cell and activating NK receptor ligation, suggesting that PI3K may follow Syk in this cascade (51, 52) (**Figure 8**). Indeed, upon NK receptor ligation, Syk activity leads to the consecutive activation of PI3K, Rac1, PAK1, MEK, and ERK (52). In addition to its role at this stage, it is possible that Syk may be involved at later stages of this process. For example, Syk associates with the cytosplasmic tails of integrin β subunits and is critical for the induction of ROS production and degranulation in response to integrin ligation in neutrophils (27, 30).

The efficacy of the PI3K inhibitor LY294002 and the SHIP1 inhibitor 3-AC demonstrate the importance of phosphatidylinositol (PtdIns) signaling in regulating CD11b recruitment to the cell surface. Acting in concert, a class I PI3K and SHIP1 would generate the PtdIns species PtdIns (3, 4)P_2_, which has previously been found to be essential for the full activation of certain pleckstrin homology (PH) domain–containing proteins, such as Akt and lamellipodin (53, 54). PtdIns species are also important regulators of Btk (PtdIns[3,4,5]P_3_) and the NADPH oxidase subunits p47phox (PtdIns[3,4]P_2_) and p40phox (PtdIns[3]P), suggesting that PI3Ks and/or lipid phosphatases are also necessary later in this cell death process (55–58). The requirement for PtdIns (3, 4)P_2_ or PtdIns[3,4,5]P_3_ in the surface upregulation of CD11b is not yet fully understood but may involve the recruitment or activation of guanine nucleotide exchange factors (GEFs) for the RhoGTPase Rac1, such as Tiam1 or Trio, through their PH domains. The binding of these GEFs to Rac1 is disrupted by the pharmacological agent NSC-23766 in this study (59), indicating the necessity of this interaction.

The apparent lack of direct interaction between SHIP1 and Siglec-8 could be explained by the recruitment of SHIP1 to the cell membrane by Syk, Dok1, Dok3, or Shc, with which SHIP1 is known to interact (60, 61). SHIP1 may also be recruited to a signaling adapter or cell-surface receptor interacting with Siglec-8 rather than to Siglec-8 itself, including ITAM-bearing molecules, as SHIP1 has been shown to bind phosphorylated ITAMs on the TCR signaling adapter CD3ζ and the BCR adapters Igα and Igβ (62, 63). In contrast, the direct binding observed between SHP-2 and Siglec-8 in response to orthovanadate treatment may reflect a Siglec-8 signaling pathway distinct from the one that terminates in cell death. Alternatively, this interaction may have no bearing on SHP-2 function or may reveal a mechanism whereby SHP-2 activity is inhibited through sequestration of the protein. Activating mutant forms of EGFR have been found to sequester SHP-2 at the membrane, consequently inhibiting its downstream activity (64).

The use of two different pharmacological inhibitors of MEK1/2 allowed us to determine that MEK1, in particular, is essential for CD11b surface upregulation in the cell death pathway. This is not the first study to identify a role for MEK1/2 in Siglec-8-induced cell death of cytokine-primed eosinophils. Using a system in which eosinophils simultaneously undergo cytokine priming with IL-5 and Siglec-8 receptor ligation, Kano et al. determined that MEK1/2 are essential for cell death but act downstream of ROS production (26). This apparent discrepancy may be explained by the fact that IL-5 signaling itself causes the acute activation of CD11b on eosinophils (65), which may rely on an alternative pathway that does not involve MEK1, thus bypassing the need for the molecule at that stage. By priming eosinophils with IL-5 for 24 h prior to Siglec-8 ligation, we can more effectively extricate the Siglec-8 signaling pathway from that induced by IL-5. However, because our approach only identifies the earliest stage at which a particular molecule is necessary, it remains possible that MEK1 is involved in both integrin upregulation and in cell death following ROS production in our system.

Although c-Raf is considered the typical activator of MEK1, several studies have found that the Rac1 target PAK1 can also activate MEK1/ERK signaling, including in a pathway in neutrophils stimulated by immune complexes that leads to cell death (32–34). Thus, the negative results with the c-Raf inhibitor GW5047 and the positive results with the PAK1 inhibitor AZ13705339 in the prevention of Siglec-8–induced cell death together indicate that PAK1 is responsible for the activation of MEK1 and the subsequent activation of ERK1/2. ERK1/2, in turn, can activate p90S6K/RSK signaling, which has been shown to activate the actin-bundling protein L-plastin by phosphorylating it at the S5 residue (66). Importantly, the PKC inhibitor GF109203x, which prevented Siglec-8–induced cell death in this study, has also been shown to inhibit the RSK proteins at similar concentrations (67). L-plastin has been found to be phosphorylated at the S5 residue in human eosinophils in response to GM-CSF stimulation, and that phosphorylated form of L-plastin is sufficient for CD11b upregulation at the cell surface (68). That study, however, demonstrated a role for PKC activity in L-plastin phosphorylation and CD11b upregulation by using both pharmacological agents and siRNA knockdown of PKCβII, leaving open the question of whether PKC or RSK is essential in our system immediately downstream of ERK1/2. Incidentally, phosphorylation of L-plastin at this residue has been observed in human eosinophils following a brief stimulation with IL-5 as well as following anti-Siglec-8 treatment in IL-5–primed eosinophils (it was not observed in IL-5–primed eosinophils in the absence of Siglec-8 ligation in this study, nor in unprimed eosinophils following Siglec-8 ligation) (8, 69). IL-3 treatment of human eosinophils increases surface CD11b expression as well, and also activates RSK signaling (70, 71). Consistent with our finding that actin filament disassembly is required for integrin upregulation, α_v_β_3_ integrin-dependent K562 cell adhesion induced by the provision of cell-permeant L-plastin peptides is disrupted by treatment with the actin filament-stabilizing agent jasplakinolide and promoted by the actin filament-disrupting agent cytochalasin D (72). In addition, oxidative stress diminishes the actin-binding and -bundling functions of L-plastin through *S*-glutathionylation (73), potentially explaining why TBHP treatment prevented Siglec-8 ligation–induced surface upregulation of CD11b in this study—in contrast to its induction of CD11b conformational activation—and indicating the presence of a negative feedback loop within this Siglec-8 signaling pathway.

The results from the inhibition of PLC suggest that PKC itself is involved prior to the surface upregulation of CD11b. PLC causes the activation of novel PKC isozymes through the generation of the second messenger diacylglycerol (DAG) and conventional PKC isozymes through DAG and the release of Ca^2+^ stores through inositol 1,4,5-trisphosphate (IP_3_). In addition to a possible role in phosphorylating and activating L-plastin, PKC may be involved in promoting the conversion of sphingomyelin to sphingosine, a lipid that acts in the activation of PAK1 (38, 39). PKCδ activates the lysosomal enzyme acid sphingomyelinase, which acts in the first step of this metabolic process (36, 37). Consistent with this role, destabilization of both of the enzymes involved in this process, acid sphingomyelinase and acid ceramidase, with the tricyclic antidepressant desipramine prevents CD11b upregulation, conformational activation, ROS production, and cell death in response to Siglec-8 ligation (41, 42). In agreement with these results, treatment of human neutrophils with exogenous sphingomyelinase alone reduces F-actin content and promotes CD18 expression and ROS production (74).

While most of the molecules that have been found to be essential for Siglec-8-induced cell death are involved in surface integrin upregulation, Btk activity and actin polymerization play roles at later stages. Btk appears to play no role in CD11b surface upregulation in this pathway but rather is involved in the conformational activation of CD11b. In concordance with this finding, Btk was previously found to be essential for CD11b/CD18 activation and downstream functions, including adhesion, migration, and ROS production, in mouse neutrophils in a genetic knockout model (31). The precise nature of the essential role played by actin polymerization in NADPH oxidase assembly and function remains unclear. However, studies have found in cell-free systems that actin enhances NADPH oxidase activity, the NADPH oxidase subunit p47^phox^ binds preferentially to filamentous actin when unmasked, and agents that cause actin depolymerization accelerate NADPH oxidase deactivation (75, 76).

The signaling cascade that has emerged from studies of Siglec-8 signaling is surprising given its cytoplasmic signaling motifs. Siglec-8 bears an ITIM and an ITSM and was therefore expected to signal through an SHP-1/2–dependent signaling pathway. Indeed, disruption of the ITIM has been found to abrogate the inhibitory effect of Siglec-8 on FcϵRI-triggered degranulation and Ca^2+^ flux in RBL cells (18). Likewise, an intact ITIM was shown to be necessary for maximal Siglec-8 endocytosis in transfected HEK293T cells, whereas the ITSM appeared to play no role (43). However, these systems did not permit the study of cell death induction, and the necessity of other Siglec-8 domains in inducing cell death has likewise not been studied. Unexpectedly, the signaling pathway identified here is more typical of an ITAM-bearing receptor. This may suggest that Siglec-8 interacts with another receptor or signaling adapter containing an ITAM motif to transduce this signal. In support of this hypothesis, Siglec-7 has been shown to induce a form of non-apoptotic ROS-dependent cell death in U937 cells independent of its cytoplasmic domain and dependent instead on its extracellular membrane-proximal C2-set domain (77), suggesting that Siglecs may transduce such signals *via* interactions with other cell-surface receptors or signaling adapters. Notably, a Siglec-8 variant with a truncated cytoplasmic domain lacking the aforementioned signaling motifs is expressed by eosinophils; in fact, this is the isoform of Siglec-8 that was first identified (2, 3, 78). Nevertheless, the contribution of this variant to the cell death response remains unknown.

Interestingly, other ITIM-bearing receptors have been found to induce cell death upon engagement. FcγRIIb, in addition to negatively regulating BCR signaling when co-ligated with that receptor, initiates a signaling cascade involving c-Abl, Btk, and Jnk1 that leads to mitochondrial membrane potential dissipation and cell death in a Bid- and Bad-dependent but caspase-independent manner when engaged separately from the BCR (79–81). This latter effect is independent of the ITIM or, indeed, the entire cytoplasmic domain of FcγRIIb (79). In addition, engagement of FcγRII with immobilized antibody also brings about the cell death of IL-5–primed human eosinophils in a CD18-dependent manner (82), although it is unclear which subtype of the receptor is responsible for this outcome. Antibody engagement of CD300LF similarly induces caspase-independent cell death on mouse myeloid cells (83). Importantly, the fact that Siglec-8 engagement-induced cell death is independent of the Fc region of the engaging antibody indicates that this does not reflect inadvertent engagement of an Fc receptor, including FcγRIIb. The elements shared between these receptor-mediated cell death pathways are intriguing and may indicate the existence of a more general pathway whereby ITIM-bearing receptors can promote cell death upon aggregation. This study identifies an unanticipated signaling cascade initiated directly by Siglec-8 engagement in cytokine-primed eosinophils and further challenges the assumption that inhibitory Siglecs—and, by extension, other ITIM-bearing receptors—effect their functions purely *via* SHP-1/2–mediated signaling pathways.

## Data Availability Statement

The data supporting this publication are available at ImmPort (https://www.immport.org/) under study accession SDY1875.

## Ethics Statement

The studies involving human participants were reviewed and approved by the Institutional Review Board of Northwestern University Feinberg School of Medicine. The patients/participants provided their written informed consent to participate in this study.

## Author Contributions

DC, YC, and JO’S performed experiments and analyzed results. YC purified all human eosinophils from peripheral blood. BB and JO’S secured funding for and managed the project. DC, BB, and JO’S planned the study. JO’S wrote the manuscript with input from all authors. All authors contributed to the article and approved the submitted version.

## Funding

This work was supported by grants from the National Heart, Lung, and Blood Institute (P01HL107151 to BB) and the National Institute of Allergy and Infectious Disease (U19AI136443 to BB and U19AI070535 subaward 107905120 to JO’S).

## Conflict of Interest

BB received remuneration for serving on the scientific advisory board of Allakos, Inc. and owns stock in Allakos. He receives publication-related royalty payments from Elsevier and UpToDate^®^. He is a co-inventor on existing Siglec-8–related patents and thus may be entitled to a share of royalties received by Johns Hopkins University during development and potential sales of such products. BB is also a co-founder of Allakos, which makes him subject to certain restrictions under University policy. The terms of this arrangement are being managed by Johns Hopkins University and Northwestern University in accordance with their conflict of interest policies.

The remaining authors declare that the research was conducted in the absence of any commercial or financial relationships that could be construed as a potential conflict of interest.

## Publisher’s Note

All claims expressed in this article are solely those of the authors and do not necessarily represent those of their affiliated organizations, or those of the publisher, the editors and the reviewers. Any product that may be evaluated in this article, or claim that may be made by its manufacturer, is not guaranteed or endorsed by the publisher.

## References

[1] Varki A Angata T. Siglecs–the Major Subfamily of I-Type Lectins. Glycobiology (2006) 16(1):1R–27R. doi: 10.1093/glycob/cwj008 16014749

[2] Floyd H Ni J Cornish AL Zeng Z Liu D Carter KC. Siglec-8. A Novel Eosinophil-Specific Member of the Immunoglobulin Superfamily. J Biol Chem (2000) 275(2):861–6. doi: 10.1074/jbc.275.2.861 10625619

[3] Kikly KK Bochner BS Freeman SD Tan KB Gallagher KT D’Alessio KJ. Identification of SAF-2, a Novel Siglec Expressed on Eosinophils, Mast Cells, and Basophils. J Allergy Clin Immunol (2000) 105(6 Pt 1):1093–100. doi: 10.1067/mai.2000.107127 10856141

[4] Foussias G Yousef GM Diamandis EP. Molecular Characterization of a Siglec8 Variant Containing Cytoplasmic Tyrosine-Based Motifs, and Mapping of the Siglec8 Gene. Biochem Biophys Res Commun (2000) 278(3):775–81. doi: 10.1006/bbrc.2000.3866 11095983

[5] Nutku E Hudson SA Bochner BS. Mechanism of Siglec-8-Induced Human Eosinophil Apoptosis: Role of Caspases and Mitochondrial Injury. Biochem Biophys Res Commun (2005) 336(3):918–24. doi: 10.1016/j.bbrc.2005.08.202 16157303

[6] Nutku-Bilir E Hudson SA Bochner BS. Interleukin-5 Priming of Human Eosinophils Alters Siglec-8 Mediated Apoptosis Pathways. Am J Respir Cell Mol Biol (2008) 38(1):121–4. doi: 10.1165/rcmb.2007-0154OC PMC2176128 17690326

[7] Na HJ Hudson SA Bochner BS. IL-33 Enhances Siglec-8 Mediated Apoptosis of Human Eosinophils. Cytokine (2012) 57(1):169–74. doi: 10.1016/j.cyto.2011.10.007 PMC3282301 22079334

[8] Carroll DJ O’Sullivan JA Nix DB Cao Y Tiemeyer M Bochner BS. Sialic Acid-Binding Immunoglobulin-Like Lectin 8 (Siglec-8) Is an Activating Receptor Mediating Beta2-Integrin-Dependent Function in Human Eosinophils. J Allergy Clin Immunol (2018) 141(6):2196–207. doi: 10.1016/j.jaci.2017.08.013 PMC5839929 28888781

[9] Calafat J Kuijpers TW Janssen H Borregaard N Verhoeven AJ Roos D. Evidence for Small Intracellular Vesicles in Human Blood Phagocytes Containing Cytochrome B558 and the Adhesion Molecule CD11b/Cd18. Blood (1993) 81(11):3122–9. doi: 10.1182/blood.V81.11.3122.bloodjournal81113122 8098969

[10] Doody GM Justement LB Delibrias CC Matthews RJ Lin J Thomas ML. A Role in B Cell Activation for CD22 and the Protein Tyrosine Phosphatase SHP. Science (1995) 269(5221):242–4. doi: 10.1126/science.7618087 7618087

[11] Duan S Koziol-White CJ Jester WF Jr. Nycholat CM Macauley MS Panettieri RA Jr.. CD33 Recruitment Inhibits IgE-Mediated Anaphylaxis and Desensitizes Mast Cells to Allergen. J Clin Invest (2019) 129(3):1387–401. doi: 10.1172/JCI125456 PMC6391081 30645205

[12] Ikehara Y Ikehara SK Paulson JC. Negative Regulation of T Cell Receptor Signaling by Siglec-7 (P70/AIRM) and Siglec-9. J Biol Chem (2004) 279(41):43117–25. doi: 10.1074/jbc.M403538200 15292262

[13] Taylor VC Buckley CD Douglas M Cody AJ Simmons DL Freeman SD. The Myeloid-Specific Sialic Acid-Binding Receptor, CD33, Associates With the Protein-Tyrosine Phosphatases, SHP-1 and SHP-2. J Biol Chem (1999) 274(17):11505–12. doi: 10.1074/jbc.274.17.11505 10206955

[14] Avril T Freeman SD Attrill H Clarke RG Crocker PR. Siglec-5 (CD170) can Mediate Inhibitory Signaling in the Absence of Immunoreceptor Tyrosine-Based Inhibitory Motif Phosphorylation. J Biol Chem (2005) 280(20):19843–51. doi: 10.1074/jbc.M502041200 15769739

[15] Angata T Kerr SC Greaves DR Varki NM Crocker PR Varki A. Cloning and Characterization of Human Siglec-11. A Recently Evolved Signaling Molecule That Can Interact With SHP-1 and SHP-2 and Is Expressed by Tissue Macrophages, Including Brain Microglia. J Biol Chem (2002) 277(27):24466–74. doi: 10.1074/jbc.M202833200 11986327

[16] Stefanski AL Renecle MD Rumer KK Winn VD. Siglec-6 Phosphorylation at Intracellular Tyrosine Residues Leads to the Recruitment of SHP-2 Phosphatase. Reprod Sci (2014) 21(3 Supplement):388A–9A. doi: 10.1177/1933719114528275

[17] Kano G Bochner BS Zimmermann N. Regulation of Siglec-8-Induced Intracellular Reactive Oxygen Species Production and Eosinophil Cell Death by Src Family Kinases. Immunobiology (2017) 222(2):343–49. doi: 10.1016/j.imbio.2016.09.006 PMC5154890 27682013

[18] Yokoi H Choi OH Hubbard W Lee HS Canning BJ Lee HH. Inhibition of FcepsilonRI-Dependent Mediator Release and Calcium Flux From Human Mast Cells by Sialic Acid-Binding Immunoglobulin-Like Lectin 8 Engagement. J Allergy Clin Immunol (2008) 121(2):499–505 e1. doi: 10.1016/j.jaci.2007.10.004 18036650

[19] Hansel TT Pound JD Pilling D Kitas GD Salmon M Gentle TA. Purification of Human Blood Eosinophils by Negative Selection Using Immunomagnetic Beads. J Immunol Methods (1989) 122(1):97–103. doi: 10.1016/0022-1759(89)90339-6 2547875

[20] Cao Y Shin S Carroll DJ O’Sullivan JA Bochner BS. Single-Site, Five-Year Experience With Human Eosinophil Isolation by Density Gradient Centrifugation and CD16 Immunomagnetic Negative Separation. BMC Res Notes (2020) 13(1):211. doi: 10.1186/s13104-020-05055-9 32276656 PMC7149875

[21] Nutku E Aizawa H Hudson SA Bochner BS. Ligation of Siglec-8: A Selective Mechanism for Induction of Human Eosinophil Apoptosis. Blood (2003) 101(12):5014–20. doi: 10.1182/blood-2002-10-3058 12609831

[22] Brooks R Fuhler GM Iyer S Smith MJ Park MY Paraiso KH. SHIP1 Inhibition Increases Immunoregulatory Capacity and Triggers Apoptosis of Hematopoietic Cancer Cells. J Immunol (2010) 184(7):3582–9. doi: 10.4049/jimmunol.0902844 PMC4123216 20200281

[23] Suwa A Kurama T Yamamoto T Sawada A Shimokawa T Aramori I. Glucose Metabolism Activation by SHIP2 Inhibitors via Up-Regulation of GLUT1 Gene in L6 Myotubes. Eur J Pharmacol (2010) 642(1-3):177–82. doi: 10.1016/j.ejphar.2010.06.002 20558154

[24] Hanke JH Gardner JP Dow RL Changelian PS Brissette WH Weringer EJ. Discovery of a Novel, Potent, and Src Family-Selective Tyrosine Kinase Inhibitor. Study of Lck- and FynT-Dependent T Cell Activation. J Biol Chem (1996) 271(2):695–701. doi: 10.1074/jbc.271.2.695 8557675

[25] Blake RA Broome MA Liu X Wu J Gishizky M Sun L. SU6656, a Selective Src Family Kinase Inhibitor, Used to Probe Growth Factor Signaling. Mol Cell Biol (2000) 20(23):9018–27. doi: 10.1128/mcb.20.23.9018-9027.2000 PMC86555 11074000

[26] Kano G Almanan M Bochner BS Zimmermann N. Mechanism of Siglec-8-Mediated Cell Death in IL-5-Activated Eosinophils: Role for Reactive Oxygen Species-Enhanced MEK/ERK Activation. J Allergy Clin Immunol (2013) 132(2):437–45. doi: 10.1016/j.jaci.2013.03.024 PMC4042061 23684072

[27] Mocsai A Zhou M Meng F Tybulewicz VL Lowell CA. Syk Is Required for Integrin Signaling in Neutrophils. Immunity (2002) 16(4):547–58. doi: 10.1016/S1074-7613(02)00303-5 11970878

[28] Obergfell A Eto K Mocsai A Buensuceso C Moores SL Brugge JS. Coordinate Interactions of Csk, Src, and Syk Kinases With [Alpha]IIb[beta]3 Initiate Integrin Signaling to the Cytoskeleton. J Cell Biol (2002) 157(2):265–75. doi: 10.1083/jcb.200112113 PMC2199242 11940607

[29] Van Ziffle JA Lowell CA. Neutrophil-Specific Deletion of Syk Kinase Results in Reduced Host Defense to Bacterial Infection. Blood (2009) 114(23):4871–82. doi: 10.1182/blood-2009-05-220806 PMC2786293 19797524

[30] Woodside DG Obergfell A Leng L Wilsbacher JL Miranti CK Brugge JS. Activation of Syk Protein Tyrosine Kinase Through Interaction With Integrin Beta Cytoplasmic Domains. Curr Biol (2001) 11(22):1799–804. doi: 10.1016/S0960-9822(01)00565-6 11719224

[31] Volmering S Block H Boras M Lowell CA Zarbock A. The Neutrophil Btk Signalosome Regulates Integrin Activation During Sterile Inflammation. Immunity (2016) 44(1):73–87. doi: 10.1016/j.immuni.2015.11.011 26777396 PMC5030078

[32] Frost JA Steen H Shapiro P Lewis T Ahn N Shaw PE. Cross-Cascade Activation of ERKs and Ternary Complex Factors by Rho Family Proteins. EMBO J (1997) 16(21):6426–38. doi: 10.1093/emboj/16.21.6426 PMC1170249 9351825

[33] Jiang K Zhong B Gilvary DL Corliss BC Hong-Geller E Wei S. Pivotal Role of Phosphoinositide-3 Kinase in Regulation of Cytotoxicity in Natural Killer Cells. Nat Immunol (2000) 1(5):419–25. doi: 10.1038/80859 11062502

[34] Chu JY Dransfield I Rossi AG Vermeren S. Non-Canonical PI3K-Cdc42-Pak-Mek-Erk Signaling Promotes Immune-Complex-Induced Apoptosis in Human Neutrophils. Cell Rep (2016) 17(2):374–86. doi: 10.1016/j.celrep.2016.09.006 PMC5067281 27705787

[35] McCoull W Hennessy EJ Blades K Chuaqui C Dowling JE Ferguson AD. Optimization of Highly Kinase Selective Bis-Anilino Pyrimidine PAK1 Inhibitors. ACS Med Chem Lett (2016) 7(12):1118–23. doi: 10.1021/acsmedchemlett.6b00322 PMC5150691 27994749

[36] Zeidan YH Hannun YA. Activation of Acid Sphingomyelinase by Protein Kinase Cdelta-Mediated Phosphorylation. J Biol Chem (2007) 282(15):11549–61. doi: 10.1074/jbc.M609424200 17303575

[37] Zeidan YH Wu BX Jenkins RW Obeid LM Hannun YA. A Novel Role for Protein Kinase Cdelta-Mediated Phosphorylation of Acid Sphingomyelinase in UV Light-Induced Mitochondrial Injury. FASEB J (2008) 22(1):183–93. doi: 10.1096/fj.07-8967com 17698617

[38] Bokoch GM Reilly AM Daniels RH King CC Olivera A Spiegel S. A GTPase-Independent Mechanism of P21-Activated Kinase Activation. Regulation by Sphingosine and Other Biologically Active Lipids. J Biol Chem (1998) 273(14):8137–44. doi: 10.1074/jbc.273.14.8137 9525917

[39] King CC Gardiner EM Zenke FT Bohl BP Newton AC Hemmings BA. P21-Activated Kinase (PAK1) Is Phosphorylated and Activated by 3-Phosphoinositide-Dependent Kinase-1 (PDK1). J Biol Chem (2000) 275(52):41201–9. doi: 10.1074/jbc.M006553200 10995762

[40] Letschka T Kollmann V Pfeifhofer-Obermair C Lutz-Nicoladoni C Obermair GJ Fresser F. PKC-Theta Selectively Controls the Adhesion-Stimulating Molecule Rap1. Blood (2008) 112(12):4617–27. doi: 10.1182/blood-2007-11-121111 18796635

[41] Beckmann N Sharma D Gulbins E Becker KA Edelmann B. Inhibition of Acid Sphingomyelinase by Tricyclic Antidepressants and Analogons. Front Physiol (2014) 5:331. doi: 10.3389/fphys.2014.00331 25228885 PMC4151525

[42] Elojeimy S Holman DH Liu X El-Zawahry A Villani M Cheng JC. New Insights on the Use of Desipramine as an Inhibitor for Acid Ceramidase. FEBS Lett (2006) 580(19):4751–6. doi: 10.1016/j.febslet.2006.07.071 16901483

[43] O’Sullivan JA Carroll DJ Cao Y Salicru AN Bochner BS. Leveraging Siglec-8 Endocytic Mechanisms to Kill Human Eosinophils and Malignant Mast Cells. J Allergy Clin Immunol (2018) 141(5):1774–85. doi: 10.1016/j.jaci.2017.06.028 PMC6445644 28734845

[44] Johansson MW. Activation States of Blood Eosinophils in Asthma. Clin Exp Allergy (2014) 44(4):482–98. doi: 10.1111/cea.12292 PMC4057046 24552191

[45] Borregaard N Kjeldsen L Sengelov H Diamond MS Springer TA Anderson HC. Changes in Subcellular Localization and Surface Expression of L-Selectin, Alkaline Phosphatase, and Mac-1 in Human Neutrophils During Stimulation With Inflammatory Mediators. J leukocyte Biol (1994) 56(1):80–7. doi: 10.1002/jlb.56.1.80 7517990

[46] Sengelov H Kjeldsen L Diamond MS Springer TA Borregaard N. Subcellular Localization and Dynamics of Mac-1 (Alpha M Beta 2) in Human Neutrophils. J Clin Invest (1993) 92(3):1467–76. doi: 10.1172/JCI116724 PMC288292 8376598

[47] Angata T Hayakawa T Yamanaka M Varki A Nakamura M. Discovery of Siglec-14, a Novel Sialic Acid Receptor Undergoing Concerted Evolution With Siglec-5 in Primates. FASEB J (2006) 20(12):1964–73. doi: 10.1096/fj.06-5800com 17012248

[48] Angata T Tabuchi Y Nakamura K Nakamura M. Siglec-15: An Immune System Siglec Conserved Throughout Vertebrate Evolution. Glycobiology (2007) 17(8):838–46. doi: 10.1093/glycob/cwm049 17483134

[49] Cao H Lakner U de Bono B Traherne JA Trowsdale J Barrow AD. SIGLEC16 Encodes a DAP12-Associated Receptor Expressed in Macrophages That Evolved From its Inhibitory Counterpart SIGLEC11 and has Functional and non-Functional Alleles in Humans. Eur J Immunol (2008) 38(8):2303–15. doi: 10.1002/eji.200738078 18629938

[50] Blasius AL Cella M Maldonado J Takai T Colonna M. Siglec-H is an IPC-Specific Receptor That Modulates Type I IFN Secretion Through DAP12. Blood (2006) 107(6):2474–6. doi: 10.1182/blood-2005-09-3746 PMC1895736 16293595

[51] Beitz LO Fruman DA Kurosaki T Cantley LC Scharenberg AM. SYK is Upstream of Phosphoinositide 3-Kinase in B Cell Receptor Signaling. J Biol Chem (1999) 274(46):32662–6. doi: 10.1074/jbc.274.46.32662 10551821

[52] Jiang K Zhong B Gilvary DL Corliss BC Vivier E Hong-Geller E. Syk Regulation of Phosphoinositide 3-Kinase-Dependent NK Cell Function. J Immunol (2002) 168(7):3155–64. doi: 10.4049/jimmunol.168.7.3155 11907067

[53] Li H Wu X Hou S Malek M Kielkowska A Noh E. Phosphatidylinositol-3,4-Bisphosphate and Its Binding Protein Lamellipodin Regulate Chemotaxis of Malignant B Lymphocytes. J Immunol (2016) 196(2):586–95. doi: 10.4049/jimmunol.1500630 26695371

[54] Scheid MP Huber M Damen JE Hughes M Kang V Neilsen P. Phosphatidylinositol (3,4,5)P3 is Essential But Not Sufficient for Protein Kinase B (PKB) Activation; Phosphatidylinositol (3,4)P2 is Required for PKB Phosphorylation at Ser-473: Studies Using Cells From SH2-Containing Inositol-5-Phosphatase Knockout Mice. J Biol Chem (2002) 277(11):9027–35. doi: 10.1074/jbc.M106755200 11781306

[55] Li Z Wahl MI Eguinoa A Stephens LR Hawkins PT Witte ON. Phosphatidylinositol 3-Kinase-Gamma Activates Bruton’s Tyrosine Kinase in Concert With Src Family Kinases. Proc Natl Acad Sci USA (1997) 94(25):13820–5. doi: 10.1073/pnas.94.25.13820 PMC28391 9391111

[56] Salim K Bottomley MJ Querfurth E Zvelebil MJ Gout I Scaife R. Distinct Specificity in the Recognition of Phosphoinositides by the Pleckstrin Homology Domains of Dynamin and Bruton’s Tyrosine Kinase. EMBO J (1996) 15(22):6241–50. doi: 10.1002/j.1460-2075.1996.tb01014.x PMC452447 8947047

[57] Ellson CD Gobert-Gosse S Anderson KE Davidson K Erdjument-Bromage H Tempst P. PtdIns(3)P Regulates the Neutrophil Oxidase Complex by Binding to the PX Domain of P40(Phox). Nat Cell Biol (2001) 3(7):679–82. doi: 10.1038/35083076 11433301

[58] Kanai F Liu H Field SJ Akbary H Matsuo T Brown GE. The PX Domains of P47phox and P40phox Bind to Lipid Products of PI(3)K. Nat Cell Biol (2001) 3(7):675–8. doi: 10.1038/35083070 11433300

[59] Gao Y Dickerson JB Guo F Zheng J Zheng Y. Rational Design and Characterization of a Rac GTPase-Specific Small Molecule Inhibitor. Proc Natl Acad Sci U.S.A. (2004) 101(20):7618–23. doi: 10.1073/pnas.0307512101 PMC419655 15128949

[60] de Castro RO Zhang J Groves JR Barbu EA Siraganian RP. Once Phosphorylated, Tyrosines in Carboxyl Terminus of Protein-Tyrosine Kinase Syk Interact With Signaling Proteins, Including TULA-2, a Negative Regulator of Mast Cell Degranulation. J Biol Chem (2012) 287(11):8194–204. doi: 10.1074/jbc.M111.326850 PMC3318758 22267732

[61] Lemay S Davidson D Latour S Veillette A. Dok-3, a Novel Adapter Molecule Involved in the Negative Regulation of Immunoreceptor Signaling. Mol Cell Biol (2000) 20(8):2743–54. doi: 10.1128/mcb.20.8.2743-2754.2000 PMC85490 10733577

[62] Osborne MA Zenner G Lubinus M Zhang X Songyang Z Cantley LC. The Inositol 5’-Phosphatase SHIP Binds to Immunoreceptor Signaling Motifs and Responds to High Affinity IgE Receptor Aggregation. J Biol Chem (1996) 271(46):29271–8. doi: 10.1074/jbc.271.46.29271 8910587

[63] Manno B Oellerich T Schnyder T Corso J Losing M Neumann K. The Dok-3/Grb2 Adaptor Module Promotes Inducible Association of the Lipid Phosphatase SHIP With the BCR in a Coreceptor-Independent Manner. Eur J Immunol (2016) 46(11):2520–30. doi: 10.1002/eji.201646431 27550373

[64] Furcht CM Munoz Rojas AR Nihalani D Lazzara MJ. Diminished Functional Role and Altered Localization of SHP2 in Non-Small Cell Lung Cancer Cells With EGFR-Activating Mutations. Oncogene (2013) 32(18):2346–55, 55 e1-10. doi: 10.1038/onc.2012.240 22777356 PMC3727284

[65] Han ST Mosher DF. IL-5 Induces Suspended Eosinophils to Undergo Unique Global Reorganization Associated With Priming. Am J Respir Cell Mol Biol (2014) 50(3):654–64. doi: 10.1165/rcmb.2013-0181OC PMC4068932 24156300

[66] Lommel MJ Trairatphisan P Gabler K Laurini C Muller A Kaoma T. L-Plastin Ser5 Phosphorylation in Breast Cancer Cells and *In Vitro* Is Mediated by RSK Downstream of the ERK/MAPK Pathway. FASEB J (2016) 30(3):1218–33. doi: 10.1096/fj.15-276311 26631483

[67] Roberts NA Haworth RS Avkiran M. Effects of Bisindolylmaleimide PKC Inhibitors on P90rsk Activity *In Vitro* and in Adult Ventricular Myocytes. Br J Pharmacol (2005) 145(4):477–89. doi: 10.1038/sj.bjp.0706210 PMC1576162 15821757

[68] Pazdrak K Young TW Straub C Stafford S Kurosky A. Priming of Eosinophils by GM-CSF Is Mediated by Protein Kinase CbetaII-Phosphorylated L-Plastin. J Immunol (2011) 186(11):6485–96. doi: 10.4049/jimmunol.1001868 PMC3100773 21525390

[69] Mosher DF Wilkerson EM Turton KB Hebert AS Coon JJ. Proteomics of Eosinophil Activation. Front Med (Lausanne) (2017) 4:159. doi: 10.3389/fmed.2017.00159 29034237 PMC5626809

[70] Esnault S Johansson MW Kelly EA Koenderman L Mosher DF Jarjour NN. IL-3 Up-Regulates and Activates Human Eosinophil CD32 and Alphambeta2 Integrin Causing Degranulation. Clin Exp Allergy (2017) 47(4):488–98. doi: 10.1111/cea.12876 PMC5378663 28000949

[71] Esnault S Kelly EA Shen ZJ Johansson MW Malter JS Jarjour NN. IL-3 Maintains Activation of the P90s6k/RPS6 Pathway and Increases Translation in Human Eosinophils. J Immunol (2015) 195(6):2529–39. doi: 10.4049/jimmunol.1500871 PMC4561194 26276876

[72] Wang J Chen H Brown EJ. L-Plastin Peptide Activation of Alpha(V)Beta(3)-Mediated Adhesion Requires Integrin Conformational Change and Actin Filament Disassembly. J Biol Chem (2001) 276(17):14474–81. doi: 10.1074/jbc.M007324200 11278342

[73] Dubey M Singh AK Awasthi D Nagarkoti S Kumar S Ali W. L-Plastin S-Glutathionylation Promotes Reduced Binding to Beta-Actin and Affects Neutrophil Functions. Free Radic Biol Med (2015) 86:1–15. doi: 10.1016/j.freeradbiomed.2015.04.008 25881549

[74] Feldhaus MJ Weyrich AS Zimmerman GA McIntyre TM. Ceramide Generation *In Situ* Alters Leukocyte Cytoskeletal Organization and Beta 2-Integrin Function and Causes Complete Degranulation. J Biol Chem (2002) 277(6):4285–93. doi: 10.1074/jbc.M106653200 11706024

[75] Tamura M Itoh K Akita H Takano K Oku S. Identification of an Actin-Binding Site in P47phox an Organizer Protein of NADPH Oxidase. FEBS Lett (2006) 580(1):261–7. doi: 10.1016/j.febslet.2005.11.080 16375898

[76] Tamura M Kanno M Endo Y. Deactivation of Neutrophil NADPH Oxidase by Actin-Depolymerizing Agents in a Cell-Free System. Biochem J (2000) 349(Pt 1):369–75. doi: 10.1042/0264-6021:3490369 PMC1221158 10861249

[77] Mitsuki M Nara K Yamaji T Enomoto A Kanno M Yamaguchi Y. Siglec-7 Mediates Nonapoptotic Cell Death Independently of Its Immunoreceptor Tyrosine-Based Inhibitory Motifs in Monocytic Cell Line U937. Glycobiology (2010) 20(3):395–402. doi: 10.1093/glycob/cwp195 20032046

[78] Aizawa H Plitt J Bochner BS. Human Eosinophils Express Two Siglec-8 Splice Variants. J Allergy Clin Immunol (2002) 109(1):176. doi: 10.1067/mai.2002.120550 11799386

[79] Pearse RN Kawabe T Bolland S Guinamard R Kurosaki T Ravetch JV. SHIP Recruitment Attenuates Fc Gamma RIIB-Induced B Cell Apoptosis. Immunity (1999) 10(6):753–60. doi: 10.1016/s1074-7613(00)80074-6 10403650

[80] Carter NA Harnett MM. Dissection of the Signalling Mechanisms Underlying FcgammaRIIB-Mediated Apoptosis of Mature B-Cells. Biochem Soc Trans (2004) 32(Pt 6):973–5. doi: 10.1042/BST0320973 15506939

[81] Tzeng SJ Bolland S Inabe K Kurosaki T Pierce SK. The B Cell Inhibitory Fc Receptor Triggers Apoptosis by a Novel C-Abl Family Kinase-Dependent Pathway. J Biol Chem (2005) 280(42):35247–54. doi: 10.1074/jbc.M505308200 16115887

[82] Kim JT Schimming AW Kita H. Ligation of Fc Gamma RII (CD32) Pivotally Regulates Survival of Human Eosinophils. J Immunol (1999) 162(7):4253–9. 10201955

[83] Can I Tahara-Hanaoka S Hitomi K Nakano T Nakahashi-Oda C Kurita N. Caspase-Independent Cell Death by CD300LF (MAIR-V), an Inhibitory Immunoglobulin-Like Receptor on Myeloid Cells. J Immunol (2008) 180(1):207–13. doi: 10.4049/jimmunol.180.1.207 18097021

