# Supplementary material for: Siglec-8 Signals Through a Non-Canonical Pathway to Cause Human Eosinophil Death In Vitro
Source: Front Immunol. 2021 Oct 11;12:737988. doi: 10.3389/fimmu.2021.737988 (PMC8549629; doi:10.3389/fimmu.2021.737988)
Supplement: Supplementary file 1 [file DataSheet_1.docx]

Supplementary Material

# Materials and Methods

## Co-immunoprecipitation assays to detect protein tyrosine phosphatase-Siglec-8 interactions

Protein-protein interactions following Siglec-8 activation were assessed using co-immunoprecipitation. Following overnight culture with rhIL-5, eosinophils (5×10^6^) were incubated with 100 μM sodium orthovanadate (Sigma, St. Louis, MO) for 15 min at 37°C and protein was isolated using standard RIPA buffer that included a cocktail of protease and phosphatase inhibitors (Thermo Scientific). Protein G agarose fast flow beads (Millipore) were coupled with 10 μM anti-SHP-1 (Millipore), anti-SHP-2 (BD Biosciences), or anti-SHIP antibody (Abcam, Cambridge, MA) for 1 h at 4°C. Protein lysates were added to the coupled beads and incubated for 2 h at 4°C. Following incubation, beads were washed three times using 1x TBS buffer. To elute captured proteins from the beads, 2x Laemmli buffer (Bio-Rad) was added and beads were boiled at 95°C for 5 min. Following protein gel electrophoresis, membranes were incubated overnight with a cocktail of mouse anti-Siglec-8 mAbs (1 μg/mL each of mAb 2C4, 4F11 [both recognizing non-overlapping sites on domain 1] and 1H10 [recognizing domain 3]; the latter two kindly provided by Dr. Nenad Tomasevic, Allakos, Inc, Redwood City, CA). Specific binding of these antibodies was detected as above using the Odyssey Imaging System.


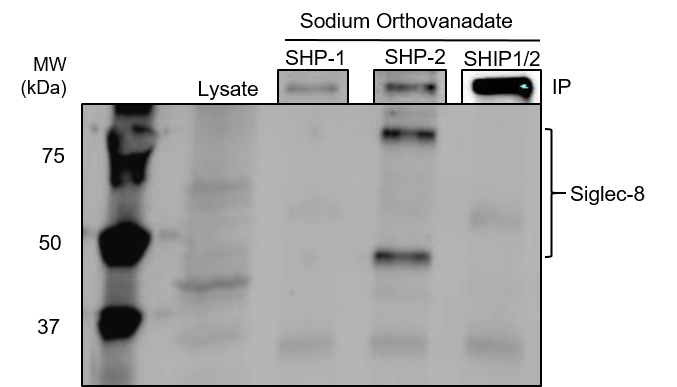


**Supplementary Figure 1.** SHP-2 associates with phosphorylated Siglec-8, but no association is observed with SHIP1. Eosinophils (5×10^6^ per condition) were primed overnight with IL-5 and incubated with 100 µM sodium orthovanadate for 15 min prior to lysis. SHP-1, SHP-2, and SHIP1/2 were immunoprecipitated from the lysates, the immunoprecipitated proteins were separated by SDS-PAGE and transferred to PVDF membranes, and the phosphatases and Siglec-8 were detected using specific mAbs.

**

**

**Supplementary Figure 2.** Treatment of IL-5–primed eosinophils with anti-Siglec-8 F(ab′)_2_ induces cell death via a pathway that, like the intact anti-Siglec-8 mAb, is dependent on Syk (OXSI-2), PI3K (LY294002), and PLC (U73122) activities. U73343 was used as an analog control for U73122. Eosinophils were primed with IL-5 and pretreated with the indicated pharmacological inhibitors for 30 min prior to treatment with anti-Siglec-8 (red bars) or isotype control mAb (blue bars) for 18–24 hr (whole IgG1, solid bars; F(ab′)_2_, bars with yellow diagonal stripes). Cell viability was then assessed by annexin V and DAPI staining by flow cytometry and normalized to that of untreated control samples. Data represent means ± standard deviations of 3 independent experiments. ****, p<0.0001 relative to the isotype control within that pharmacological inhibitor treatment group.
